# Langerhans cell histiocytosis of the skull in 23 children

**DOI:** 10.1186/s40001-024-01727-5

**Published:** 2024-02-17

**Authors:** Min Wei, Wenbin Jiang, Rui Wang, Bo Xiao, Qijia Zhan

**Affiliations:** Department of Neurosurgery, Shanghai Children’s Hospital, Shanghai Jiao Tong University, Luding Rd. 355, Shanghai, People’s Republic of China

**Keywords:** Skull, Langerhans cell histiocytosis, Clinical manifestations, Diagnosis, Treatment

## Abstract

**Objective:**

To explore the clinical features, diagnosis, treatment and prognosis of Langerhans cell histiocytosis (LCH) of the skull in children.

**Methods:**

This study retrospectively summarized the clinical manifestations, treatment methods and follow-up status of children with skull LCH who were admitted to the Department of Neurosurgery of Shanghai Children’s Hospital from January 2014 to June 2021.

**Results:**

A total of 23 patients confirmed by histology as LCH received hospitalization treatment, including 14 males and 9 females, aged (5.76 ± 3.86) years old. The clinical manifestations were mostly incidentally discovered head masses that gradually enlarged (19 cases, 82.61%). Only 2 cases are affected by multiple systems, while the rest are affected by single systems. 9 patients were involved in multiple skull lesions, and 14 patients had local skull lesions. All patients underwent surgical intervention, with 17 patients undergoing total resection and 6 patients undergoing biopsy. 21 patients received chemotherapy after surgery. The median follow-up was 2.46 years (range 0.33–6.83 years). 21 patients had their symptoms and signs under control or even resolved, and 2 patients experienced recurrence during follow-up. The overall control rate reached 91.30%.

**Conclusion:**

Personalized treatment plans according to different clinical types. Regular outpatient follow-up is crucial to monitor disease recurrence and late effects.

## Introduction

Langerhans cell histiocytosis (LCH), formerly known as histiocytosis X, poses a challenge due to its unknown etiology, sparking ongoing debates on whether it represents a malignant process or an immune-mediated inflammatory state. Pathological Langerhans cells exhibit proliferation in various human organs [1]. While LCH lesions can manifest in different tissues, including skin, subthalamic, liver, lung, or lymphoid tissues, the disease most commonly targets bone, with the skull being the primary site of involvement [1, 2]. Despite its significance, there is a limited number of literature reports on LCH in children’s skulls, making it susceptible to misdiagnosis. The optimal therapy for LCH remains undefined. This study reviews clinical data from 23 pediatric patients with cranial LCH, systematically analyzing their clinical and imaging characteristics. Additionally, it reports treatment outcomes involving surgical resection combined with systemic intravenous chemotherapy, supplemented by a comprehensive review of relevant literature reports to gain insights into managing this rare entity.

## Materials and methods

This retrospective study collected clinical manifestations, imaging examinations, treatment methods, and follow-up data from children with skull LCH admitted to the Neurosurgery Department of Shanghai Children’s Hospital between January 2014 and June 2021. Diagnostic criteria were based on pathological and immunohistochemical results, aligning with the latest Langerhans cell proliferation guidelines from the International Organization Cell Association [3].

Based on preoperative examinations, the size and location of the lesion and the extent of affected bone were determined to formulate personalized treatment plans. LCH was categorized into single system involvement (skull only, further divided into single or multiple lesions) and multiple system involvement ( ≥ 2 systems) [4]. Surgical resection was performed for single skull lesions, with observation and follow-up for lesions less than 3 cm in diameter. Larger lesions required skull repair, and if dura mater invasion was detected during surgery, postoperative chemotherapy was administered. For cases with multiple skull parts and multisystems involved, lesion resection and biopsy were conducted, followed by chemotherapy after pathological confirmation. Follow-up assessments, including various imaging studies (chest CT, bone marrow puncture, abdominal B-ultrasound, and pituitary MRI), were conducted at regular intervals. The chemotherapy regimen adopts a combination of steroids and vincristine chemotherapy. Followed up every 3 months after surgery, and changed to every 6 months after 1 year.

Define efficacy evaluation based on patient prognosis. If the original symptoms and signs disappear, or persist without progress, and there is no new disease damage, it is defined as effective control. If the original symptoms and signs continue to worsen, or if there are new lesions, it is defined as disease progression.

## Result

Twenty-three children were treated with surgery and diagnosed pathologically as LCH (Table 1). Among them were 14 males (60.87%) and 9 females (39.13%), the male to female ratio was 1.56:1.0; the age at admission was 1 year–12 years and 3 months, with an average of (5.76 ± 3.86) years old, among which, > 1–3 years old 9 cases (39.13%), 3 cases (13.04%) 3–6 years old, 11 cases (47.83%) ≥ 6 years old. According to the LCH classification of Greenberger et al. only 2 cases are affected by multiple systems, while the rest are affected by single systems. 9 patients were involved in multiple skull lesions, and 14 patients had local skull lesions. [5].

**Table 1:** Detailed baseline of the patients

Characteristic	*n* = 23 (%)
Sex	
Male	14 (60.87%)
Female	9 (39.13%)
Age (mean ± SD, years)	5.76±3.86
System involved	
Multi-system	2 (8.70%)
Spinal lesion and hepatosplenomegaly	1 (4.35%)
Pituitary lesion	1 (4.35%)
Single system	21 (91.30%)
Skull lesion	
Multifocal	9 (39.13%)
Single focal	14 (60.87%)
Frontal bone	8 (34.78%)
Parietal bone	3 (13.04%)
Temporal bone	3 (13.04%)
Operation choice	
Biopsy	7 (30.43%)
Total excision	16(69.57%)
Postoperative observation or treatment	
Observation	2 (8.70%)
Chemotherapy	21 (91.30%)
Follow-up (mean ± SD, years)	2.46±2.06
Recurrences	2 (8.70%)

### Clinical manifestations

The most common symptom of LCH is a gradually enlarging tender mass, which can touch the damaged edge of the skull when it gradually becomes soft. There was no history of trauma before the onset. Among them, 19 cases (82.61%) were treated with head masses, followed by other systemic symptoms (diabetes insipidus and hepatosplenomegaly).

### Imaging examination

All children underwent cranial CT examination. Skull osteolytic bone destruction was seen. In this group, there were 8 cases of single skull damage involving the frontal bone, 2 cases of the occipital bone, 2 cases of the temporal bone, 3 cases of the parietal bone, and 8 cases of frequent skull damage. In 15 cases, only the outer plate of the skull was destroyed, and 8 cases of the most common cases of the skull were the destruction of both the inner and outer plates of the skull. The diameter of skull lesions (the outer plate is the standard) was 1.5 to 6.5 cm, of which 2 cases had lesions greater than 3 cm in diameter. Postoperative comprehensive evaluation showed that one case had spinal lesions on CT and hepatosplenomegaly on ultrasound, while another case had pituitary lesions on MRI.

### Surgical methods

**Fig. 1:**
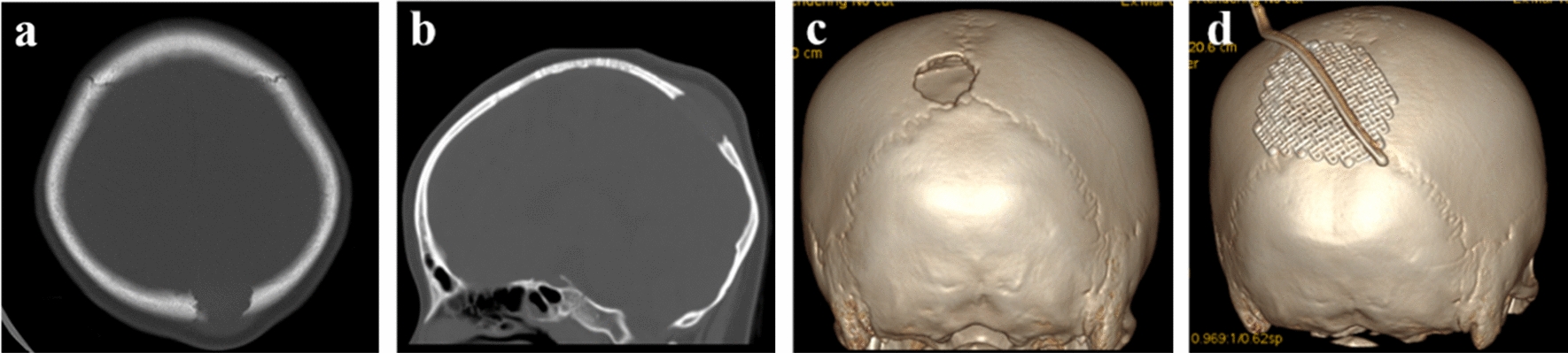
Nine years and 4 months old girl, hospitalized due to the discovery of a subcutaneous mass on the left top for 1 month. **a**–**c** The skull bone defect and a subcutaneous mass on the left top. **d**. Titanium plate is trimmed and the skull defect is repaired

All 16 cases in this group underwent total lesion resection, of which 2 cases were found to have dural invasion, and the remaining 7 cases underwent biopsy of the lesions. During the surgery, the diseased tissue was removed and the surrounding abnormal bone tissue was bitten. Among them, individual lesions with a diameter exceeding 3 cm were repaired with titanium mesh for skull repair (Fig. 1). The intraoperative lesion is a grayish yellow and reddish brown granulation like tissue beneath the periosteum, with a brittle texture, rich blood supply, and no obvious capsule. The edge of the skull defect is osteoporotic, and granulation like tissue can be seen embedded in the plate barrier. Under the microscope, diffuse proliferation of Langerhans cells can be observed, with infiltration of eosinophils, neutrophils, lymphocytes, and multinucleated giant cells. Immunohistochemistry confirmed its pathological diagnosis.

### Chemotherapy

Twenty-one children received chemotherapy after comprehensive evaluation, while the remaining 2 children with single focal lesion of the skull (without invasion of the dura mater) underwent outpatient follow-up. All cases in this study were treated with chemotherapy regimen of vinblastine (VBL) and prednisone (PRED). The specific course of treatment is 6–12 weeks of initial treatment (PRED oral and VBL weekly injection), followed by maintenance treatment (PRED given on the 1–5th day of every 3 weeks, VBL given on the 1st day), with a total treatment time of 12 months.

### Follow-up

**Fig. 2:**
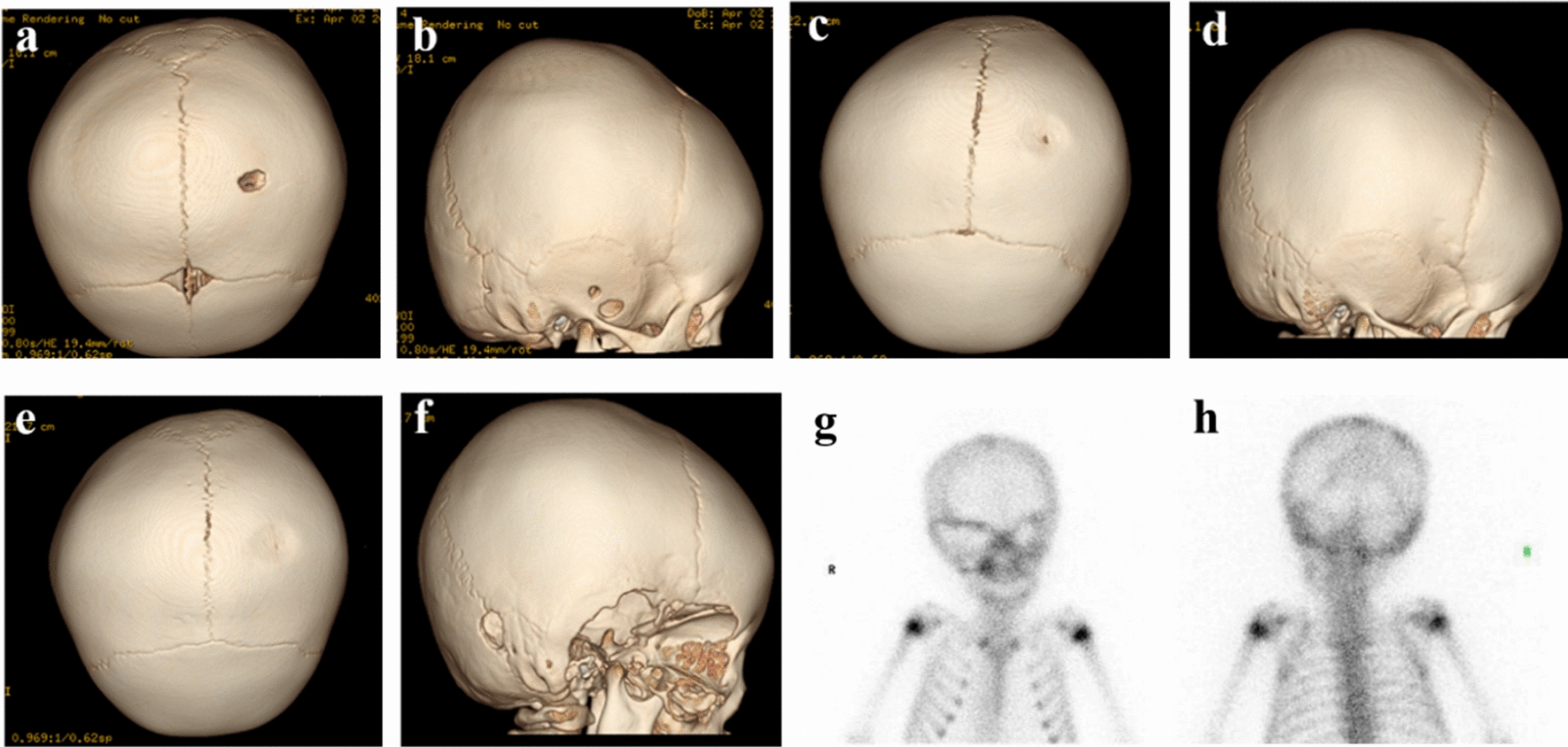
One year old girl was admitted to the hospital for *pale complexion, fatigue more than 1 month*. **a**, **b** The left parietal bone defect, 9.5 × 5.7 mm^2^, the right temporal bone defect, 5.7 × 10.2 mm^2^, 5.4 × 4.5 mm^2^.**c**, **d** The left parietal bone is nearly healed, and the right temporal bone lesions disappeared after chemotherapy. **e,** **f** The child did not receive regular chemotherapy due to COVID-19 infection, and new temporal bone lesions appeared 2.1 years after the operation, and the right temporal bone lesion was significantly expanded. **g**, **h** That the right periorbital bone lesions can be seen on SPECT

During the follow-up period, 21 patients had their symptoms and signs under control or even resolved, while 2 patients experienced recurrence, one of whom had multiple lesions that recurred during the quiescent period after routine chemotherapy after surgery. The other patient with multiple systemic involvement did not receive regular chemotherapy due to COVID-19 infection (Fig. 2). A new temporal bone lesion was discovered 2.1 years later and is currently being followed up in the outpatient department. The overall control rate reached 91.30%.

## Discussion

LCH could be onset at all ages, often in children and young people [2], occasionally in adults, and reports of onset in the neonatal period [6]. The incidence of LCH in children is about 3-5 cases per million [1], and it is more common in male than female, with a 2:1 ratio [7], which is close to our research results(1.56:1). Bone involvement is common, and it can be unifocal or multifocal [2]. In both adults and children, the skull and chest wall are the most commonly affected bones [8]. A 10-month-old mandible was reported in the literature, and no recurrence was identified after 5 years of follow-up following surgery [5]. Cho et al. hypothesize that LCH is formed from eosinophil infiltration caused by CCL-1/eotaxin-1 [8]. According to new research, LCH is a clonal tumor that arises from the unregulated proliferation and accumulation of immature myeloid dendritic cells produced from bone marrow [9].

LCH of skull involved the skull cover more common than the skull base [10]. The literature reported that the parietal and frontal bones are more common in the affected area, followed by the temporal bone, and the occipital bone is the least common, similar to the results of this study [10]. The main complaints of this group of cases were mostly accidental skull masses. In this study, 19 cases (82.61%) were treated with head masses, and the boundaries of tenderness were mostly unclear. It is believed that the clinical diagnosis of skull LCH mainly relies on radiological examinations [11]. The CT scan image generally has single or multiple, round to oval osteolytic lesions with sharp borders giving a punched out appearance with indistinct bone margins in association with a homogeneous soft tissue mass. A “Double-contour” or “beveled edge” appearance due to involvement of both the inner and outer tables may be seen [12]. The diagnostic criteria of LCH that Langerhans cell-specific protein is the gold standard for diagnosis were defined by The Histiocyte Society Writing group in 1787 [13].

Due to the variable natural history of LCH, the current treatment is still controversial. The prognosis of LCH depends on the age of onset, the extent of organ involvement and the degree of organ dysfunction. We advocate personalized treatment plans, and use different treatment methods according to different clinical types, lesion areas, and characteristics of the affected parts.

Regarding the treatment of skull LCH, the following key issues are worth exploring!

- *Biopsy versus total resection* For single system local lesions, for single system multiple lesions with similar locations, total resection is chosen. In addition, biopsy can be chosen for other classification of lesions. The selection of biopsy for multiple lesions should follow such principles: larger lesions that do not affect aesthetics (within the hairline), avoiding important blood vessels and are easily obtainable. Teranishi Y reported a case of isolated LCH of the occipital condyle received the minimally invasive biopsy with the aid of the navigation system [14]. A single localized lesion may shrink spontaneously [13].
- *Chemotherapy or not* Postoperative chemotherapy is required for patients with accumulated dura mater in a single system single skull lesion, otherwise chemotherapy is not necessary. Chemotherapy is required for other multiple lesions and children with multiple system involvement. Observation is only considered to be a viable option for the treatment of solitary skull masses with imaging characteristics [15]. Our center has chosen the VP regimen, which is currently the standard chemotherapy regimen internationally [16]. For MS-LCH patients who received treatment outside of the controlled clinical trial, an initial treatment period of 6–12 weeks (oral steroids and weekly injection of VBL) was included, followed by PRED/VBL pulses every 3 weeks for a total treatment time of 12 months. The main advantages of this regimen are the solid evidence for its activity in LCH, its acceptable toxicity, its applicability in an outpatient setting, drug availability, and the relatively low price [17]. For high-risk LCH (i.e., refractory LCH) that is ineffective with VP regimen chemotherapy, we mainly use high-dose combination chemotherapy such as cladribine (2-CdA), cytarabine, and clofarabine for rescue treatment [18, 19].
- *Radiotherapy or not* Radiotherapy has been suggested as an adjuvant treatment for local control of long bone lesions [20]. Zhang XH hold that a low dosage of radiotherapy is also an effective treatment for the LCH lesions, and in their series, a low dosage of radiotherapy allowed for a complete remission of an occipital lesion and a second time remission of recurrent orbital LCH lesions [21]. However, radiotherapy is generally not recommended for LCH in childhood due to the long term sequelae [22]. There is not enough publication literature on multicenter prospective trial to confirm these findings and determine guidelines or indications for radiotherapy to manage these cases. Therefore, none of the children in our center received radiotherapy.
- *Other therapeutics* Immunotherapy can be supplemented at the same time [23]. Targeted therapy is still experimental and can only be used as an additional treatment, while alternative therapy, the optimal dose, time and duration of treatment have yet to be determined [8]. There have been literature reports that the use of dabrafenib has achieved significant therapeutic effects in three LCH patients with BRAF-V600E gene positive mutations [24]. In addition, complications such as diabetes insipidus, short stature, hypothyroidism, hypoadrenal function, and hypogonadism may require treatment with appropriate hormones. Hearing loss and musculoskeletal disabilities should be treated appropriately [25].

It must be noted that for children with LCH, regular outpatient follow-up is crucial to monitor disease recurrence and late effects. 2 patients in this study experienced postoperative recurrence. One of the cases recurred again in the stationary period after regular chemotherapy after surgery, and the other was not scheduled due to infected COVID-19. A new temporal bone lesion appeared 2.1 years later. The recurrence rate of this study is 8.70%, which is close to the literature reports (1.6–25%) [26].

The current study has several limitations. In addition to its retrospective nature, the study group was small and heterogeneous, and duration of follow-up in our cases was still relatively short. Our conclusions require to be justified in the following studies with more cases, longer follow-up, and better with a control group.

In summary, personalized treatment plans according to different clinical types of LCH. Regular outpatient follow-up is crucial to monitor disease recurrence and late effects.

## Data Availability

The data that support the findings of this study are available from the corresponding author upon reasonable request.
